# The Application of Bioelectrical Impedance Analysis Phase Angle in Cardiac Surgery

**DOI:** 10.3390/nu17111914

**Published:** 2025-06-02

**Authors:** Joanna Popiolek-Kalisz, Grzegorz Kalisz, Michal Zembala

**Affiliations:** 1 Department of Clinical Dietetics, Medical University of Lublin, 20-059 Lublin, Poland; 2 Department of Cardiology, Cardinal Wyszynski Hospital in Lublin, 20-718 Lublin, Poland; 3 Department of Bioanalytics, Medical University of Lublin, 20-059 Lublin, Poland; grzegorz.kalisz@umlub.pl; 4 Department of Cardiac Surgery and Transplantology, John Paul II Catholic University of Lublin, 20-950 Lublin, Poland

**Keywords:** cardiac surgery, bioelectrical impedance analysis, phase angle, malnutrition, nutritional status, cardiovascular surgery

## Abstract

**Introduction:** Malnutrition is a recognized risk factor for unfavorable clinical outcomes and complications in cardiovascular and surgical patients. Nutritional status can be assessed with various methods, and the phase angle (PA) derived from bioelectrical impedance analysis is one of the most reliable parameters for that purpose. **Methods:** The aim of this narrative review was to present the available data regarding PA application in cardiac surgery. After careful analysis of PubMed and Scopus databases, 21 articles were included in the final analysis. **Results:** PA is a parameter that can be used for nutritional status assessment in a cardiac surgery environment. It is suggested that it is more accurate than other criteria in malnutrition identification in this group of patients. The available data shows its association with mortality, length of intensive care unit stay, and hospital stay. It can also predict adverse events such as blood transfusion or mechanical ventilation. **Conclusions:** The available data underscores the usefulness of PA in preoperative risk assessment and post-procedural monitoring. PA could be introduced in everyday clinical assessment in cardiac surgery.

## 1. Introduction

Malnutrition is a recognized risk factor for unfavorable clinical outcomes and complications in cardiovascular patients. It is associated with higher cardiovascular risk [1,2,3]. It can also result in a worse prognosis for chronic heart failure [4] and higher mortality in the course of acute decompensated heart failure [5,6]. It is associated with a longer length of hospitalization in patients with acute myocardial infarction [7] and higher mortality in cardiovascular patients [8]. Its role was also proven in semi-invasive procedures such as percutaneous coronary interventions and cardiac implantable electronic device implantations [9,10,11]. Clinical nutrition was initially developed in the field of surgery, where malnutrition was linked to the adverse outcomes, and as such identified as a risk factor, e.g., in gastrointestinal and oncological surgery [12,13]. This is why nutritional status assessment is crucial for cardiovascular patients and those undergoing surgery.

Malnutrition can be diagnosed with various methods, including parameters such as body mass and body mass index (BMI), recognized descriptive scales, and more complex methods, such as body composition assessment. Body mass is the most available option for nutritional status assessment. It serves as the basis for the calculation of BMI, which, according to the World Health Organization, can be interpreted as underweight when it is lower than <18.5 kg/m^2^ [14]. Due to the low cost and minimal competencies required for its measurement, BMI is a primary parameter used for malnutrition diagnosis according to the European Society for Clinical Nutrition and Metabolism (ESPEN) [15]. Nutritional risk can also be assessed with available scales, such as Nutritional Risk Screening 2002 or Subjective Global Assessment [16,17]. On one hand, it requires access to reliable nutritional history, but on the other hand, it provides a deeper insight into patients’ predicted risk [16,17]. This form of nutritional status screening is compulsory in hospitals in several countries, including Poland.

Despite the fact that BMI and body mass remain widely used, they cannot distinguish between fat mass and muscle mass, or assess shifts between fluid compartments, which are particularly important factors in cardiac surgery patients. Inflammation, edema, and catabolic conditions may mask undernutrition, and limit the clinical utility of anthropometric measurements alone. As a result, growing interest in the incorporation of functional biomarkers, such as body composition, has been observed. Body composition provides information about body compartments and their proportions to reflect nutritional status better than body mass alone. Moreover, the measurement is quick and is independent of the patient’s individual reliability, which is particularly useful in elderly patients [18]. Furthermore, regarding the ESPEN guidelines, malnutrition is defined as body composition changes leading to physical and mental activity impairment which affects the treatment outcome [15]. This is why nutritional status assessment based on body composition analysis, when available, is the most accurate approach. Body composition can be assessed with various methods, including dual-energy X-ray absorptiometry, computer tomography, and magnetic resonance imaging, but bioelectrical impedance analysis (BIA) is the most often used in clinical practice.

BIA is a non-invasive method based on applying a low-intensity alternating electrical current through the body. At the return to the analyzer, the electrical current properties are changed from the initial. This results from different electrical properties of selected body compartments, due to their water content and cell membrane properties. The functional nature of the measurement provides not only body composition data, such as fat mass or body cell mass, but also raw bioelectrical parameters such as phase angle (PA). PA is the result of the phase shift of the applied electrical current. Cell membranes are built of lipid bilayers, which is why they are poor conductors; however, they can store electrical charge, which makes them bioelectrical capacitors. The well-nourished cells can build properly formed membranes that act as good capacitors. This way, healthy cells present high reactance and higher PA than malnourished ones. PA measured at 50 kHz is a recognized standard for nutritional status assessment [18,19]. The potential functional changes at the cellular level can proceed with the full body composition abnormalities, which is why PA is a better parameter for nutritional status assessment at the cellular level [20,21]. The normal range of this parameter is 5–7 degrees [22]. PA is calculated as the arctangent of reactance-to-resistance ratio and reflects both the amount and quality of soft tissue. As already mentioned, in well-hydrated, non-edematous tissue, high cellularity and intact cell membranes produce higher reactance and thus a higher PA. In contrast, tissue injury, inflammation, or malnutrition reduces membrane capacitance, lowering reactance and leading to a lower PA. This biophysical principle supports PA’s use as a potential marker of tissue integrity and nutritional status at the cellular level. Moreover, unlike widely used body composition parameters such as fat-free mass, skeletal muscle mass, or body cell mass, which are based on regression equations and predictive models built into the analytical software of specific BIA devices, PA is a raw measurement obtained directly from impedance data. These formula-based estimates may vary between manufacturers and device models, leading to limited comparability across studies and reliability in individual patients. This is why PA is less susceptible to inter-device variability, which enhances its reliability and supports its clinical utility in the perioperative and critical care settings, where reliable methods are needed.

In recent years, PA has also gained attention beyond nutritional assessment alone, with it also being used as a prognostic marker in different clinical conditions, including oncology, nephrology, and intensive care [3,23,24,25,26]. Moreover, its role in cardiology has also been emerging, due to its ability to reflect both hydration status and cellular health [5,26]. As cardiac surgery patients are a particularly complex group, the integration of PA into routine evaluation could potentially lead to better clinical insight.

The aim of this narrative review was to present the available data regarding PA application in cardiac surgery.

## 2. Methods

**Figure 1:**
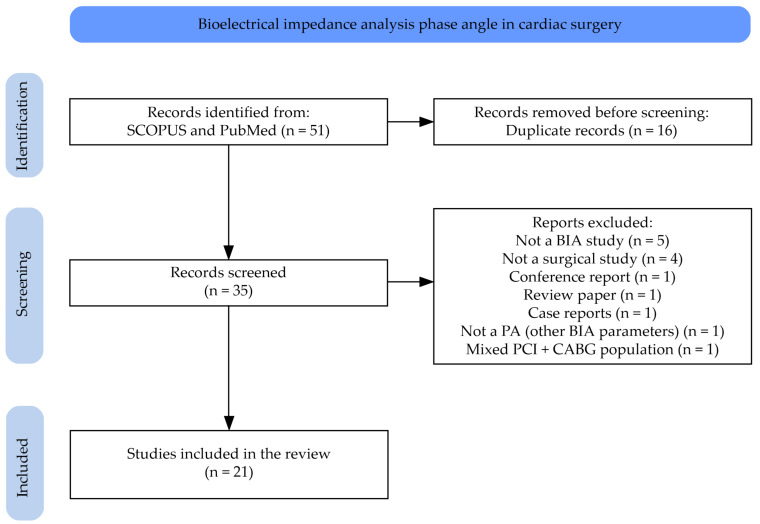
Flowchart detailing the database searches and the number of articles at each stage.

PubMed and Scopus databases were searched for all available studies that used PA in cardiac surgery patients, without any language or time limitations, using the following search: (“phase angle”) AND ((“cardiac surgery”) OR (“valve replacement”) OR (“valve surgery”) OR (“CABG”)). Purposely, patients suffering from congestive end-stage heart failure undergoing cardiac surgical procedure such as heart transplantation or/and ventricular assist device implantation were omitted, as they are often malnourished due to the severity of the primary disease. Retrieved records were independently assessed for eligibility by two trained researchers (J.P.-K. and G.K.) based on inclusion and exclusion criteria and on title and abstract screening. Disagreements were resolved by discussions involving an independent author (M.Z.) until a consensus was reached. Thereafter, the same three investigators independently reviewed the full text of potentially eligible studies to confirm their inclusion or exclusion. In total, 51 papers were initially found. Of these, 16 articles were duplicates, 1 publication was a conference report, 1 was a review article, and 1 was a case report. After abstract and full-text analyses, five articles were excluded as they did not refer to BIA PA, one article was excluded as it referred to BIA but did not provide data on PA, four articles described non-surgical populations, and one article presented mixed data on coronary artery disease patients treated with percutaneous coronary intervention and coronary artery bypass graft (CABG), but did not provide separate data on CABG patients only. After careful analysis, 21 articles were included in the final narrative review. A flowchart of the articles’ assessment is presented in Figure 1.

Although this review was conducted in the form of a narrative review, this approach was justified by the diversity and robustness of the available literature on the topic. On one hand, the included studies covered a wide range of clinical scenarios, surgical procedures, and patient populations, including both adult and pediatric cohorts, while on the other hand, stratifying the data into homogeneous subgroups would have resulted in too few studies within each category to permit meaningful quantitative synthesis. Thus, due to considerable heterogeneity in the studies’ design, endpoints, and PA measurement protocols, a systematic review or meta-analysis was not feasible. Nonetheless, the overall quality and consistency of the findings support meaningful clinical interpretation.

## 3. PA Impairment Risk Factors

As already mentioned, PA measurements can identify malnutrition before its clinical presentation. This is why PA is advised for advanced nutritional risk assessment. A prospective study of 549 cardiac surgery patients showed that the new ESPEN diagnostic criteria were not concordant with low PA observations [27]. In this study, a PA value of less than the 15th percentile of the age and gender group was set as a theoretical marker of early malnutrition. Fewer patients are classified as malnourished by the new ESPEN definition than those identified by PA, so the authors suggested that the incorporation of PA into the new ESPEN definition may aid in diagnosing the early stages of malnutrition in the field of cardiac surgery [27]. Malnutrition in cardiac surgery patients is not a rare condition, which is why it is crucial to identify the potential risk factors associated with PA decline. In 325 cardiac surgical patients, a low PA (with a cut-off value of 5.38°) was present in 29.8% and was associated with low BMI. However, the relationship with a lower handgrip strength test and with unintended weight loss or immune function was not significant [28]. Low PA was also associated with low BMI and low FFMI in this study. After multivariate analysis, a preoperative low PA emerged as a more potent indicator compared to age, gender, operative risk, C-reactive protein (CRP), albumin, NT-pro-BNP, operative procedure, cardiopulmonary bypass (CPB), and aortic cross-clamp (ACC) [28]. Interestingly, lower PA values were observed in women compared to men (50% vs. 22.2%). Moreover, in a study of 712 patients which aimed to assess the clinically relevant risk factors of malnutrition in cardiac surgery patients, low PA was present in 22.9% of patients, with a lower average PA in women and renal failure patients [29]. The analysis of disease-related risk factors of low PA indicated the following: NYHA IV class, valve pathology, renal insufficiency, body mass index, levels of hemoglobin, and CRP [29]. Moreover, the lifestyle variables that qualified as risk factors concerned the intake of food and mobility [29]. The multivariate analysis indicated that the Mini Nutritional Analysis questionnaire on lifestyle and psychological factors was more potent than PA measurements [29].

## 4. PA in Preoperative Risk Assessment

A nutritional status assessment can be performed as part of risk assessment before a surgical procedure. In the BICS (Bioimpedance in Cardiac Surgery) study, which was based on 277 patients undergoing major cardiac surgery, a lower PA after adjusting for Society of Thoracic Surgeons-predicted mortality was associated with higher mortality at 1 month, and a higher risk of overall morbidity and longer hospital length of stay at 12 months [30]. However, lower-PA patients were older, had lower BMIs, and were women. In clinical history, chronic kidney disease, malignancy, and anemia were dominating factors; interestingly, the incidence of coronary artery disease was lower. Patients with lower PA had a higher incidence of death, major adverse cardiac events, acute kidney injury, bleeding, and delirium; a longer length of stay; and higher rates of readmission. PA addition improved the results of the STS risk score for mortality; however, it is worth noting that the cut-off point was <4.5°. In the already-mentioned study of 325 cardiac surgical patients, a preoperative low PA was associated with a prolonged intensive care unit and hospital stay [28]. Moreover, in a prospective study in 342 low-operative-risk patients, low PA was also associated with higher rates and risk of postoperative morbidity in a mostly male population [31]. As a marker of malnutrition, it was evaluated as good, with malnutrition correlating with FFMI. The authors chose to use standardized PA (formula presented below) and stratification on age and gender.

$$standardized\;PA=observed\;PA-\frac{mean\;PA\;(reference)}{standard\;deviation\;PA\;(reference)}$$

A similar observation was made in a retrospective cohort study of 858 participants [32]. All-cause mortality risk was reduced in patients with higher PA. The developed predictive model was superior when it consisted of the clinical model and PA compared to physical function indicators. Each 0.1° increment in PA corresponded to a hazard ratio of 0.91 for all-cause mortality [32]. This relationship was also observed after adjustments for preoperative risk factors of postoperative morbidity. The patients with lower PA also presented a tendency to have a prolonged hospitalization (>14 days) rate, but this observation was not statistically significant. Partial discrepancies could result from the fact that this study included only low-risk patients.

Moreover, PA can be used as a risk factor for other peri-procedural complications. In an observational retrospective study which comprised 642 adult patients undergoing elective cardiac surgery, lower preoperative PA (<15th percentile) was an independent predictor of blood transfusion [33]. The importance of this parameter was highlighted with ACC time and CPB time in the univariate analysis. In the multivariate regression model, alongside PA in the <15th percentile, preoperative stroke, hemoglobin value, and CPB time were indicated as predictive factors for a postoperative RBC transfusion. The authors also concluded that hemoglobin levels at both extremes—unusually high or low—correlate with declining PA values. This suggests that red blood cell composition and structural integrity directly influence electrical conductivity, but elevated hemoglobin does not automatically guarantee a high PA [33].

To sum up, lower preoperative PA can be used as the risk factor for higher mortality and longer ICU and hospital stay. It can also predict blood transfusion risk. This is why routine nutritional status assessment with PA could identify high-risk patients. A summary of available studies that proved the usefulness of PA in cardiac surgery patients is presented in Table 1.

## 5. PA in Post-Procedural Monitoring

PA can also be used to monitor patients after surgical procedures. A historical cohort study in 204 patients showed that PA measured within the 6 h after ICU admission for postoperative care after cardiac surgery with CPB was significantly associated with in-hospital mortality or prolonged hospital length of stay [34]. This relationship was present alone and after adjustment for sex, age, BMI, or the European System for Cardiac Operative Risk Evaluation score (EuroSCORE II). However, the single-center nature of the study and the small group were declared as limitations. The authors suggest it to be relevant to combine the phase angle and hemodynamic and myocardial damage parameters. In 168 elective cardiac surgical patients, PA significantly decreased from the preoperative stage to after the procedure [40]. Measurements were performed before anesthesia and once daily, up to 7 days after hospital discharge. Furthermore, lower preoperative PA values in the patient subgroup were observed up to the 6th postoperative day. Multivariate analysis showed that higher PA was associated with lower frailty scores and LVEF. The authors also postulated associations between three out of nine intraoperative risk factors: combined surgical procedures, longer CPB, and higher fluid balance on the day of surgery. Additionally, the lowest values of PA were observed on postoperative day 2.

Patients with lower preoperative PA were older and frailer, needed more fluids, and stayed longer in the ICU. Moreover, the postoperative PA decline was independently influenced by higher fluid balances and longer CPB times [40], which could, to some extent, contribute to worse outcomes. These short-term observations were confirmed in the observational study of 130 patients undergoing cardiac surgery, where procedure-induced fluid overload was associated with slower recovery to baseline conditions [43]. Within this study, a significant change in PA was observed without returning to the baseline value [43]. Hospital functional decline was also reported by Morisawa et al. in the study on 114 patients undergoing elective cardiovascular surgery after one week [42]. The decline was measured by the Short Physical Performance Battery score, and after multivariate analysis, PA was found to be more useful in predicting the risk for hospital-acquired functional decline than preoperative physical function and nutritional status in older patients undergoing cardiovascular surgery [42]. It was also indicated that PA is associated with undernutrition and a prolonged duration of mechanical ventilation.

In a post hoc analysis of 127 well-nourished patients undergoing “on-pump” elective cardiac surgery, participants with longer hospital stays (median 14.5 days, 11.53% hospital mortality) presented a higher magnitude of PA change compared to early-recovery patients (median 8 days, 0% mortality) [38]. Measurements were made twice: before surgery and after seven days. Patients were divided into two groups: early recovery (intubation time < 24 h) and late recovery (intubation time > 24 h). The results were calculated as the magnitude of change (Δ) for each patient. In the late-recovery group, the measurements after surgery revealed changes in extracellular water (ECW) and PA, representing the cellular membrane integrity of the endothelium. Basic PA change was not significant; however, ΔPA, and also ΔECW, ΔSMML (skeletal muscle mass loss), ΔHGS (handgrip strength), and ΔMUAC (mid-upper arm circumference), differed significantly between groups [38]. It is worth noting that baseline measurements were similar in both groups. Moreover, in a cohort of 179 cardiac surgery patients, significant impairments of PA and handgrip strength were also observed 7 days after the procedure [35]. They were associated with a prolonged hospital length of stay. Moreover, among the investigated parameters, postoperative low PA was the most potent predictor of prolonged hospital length of stay and in-hospital mortality, not the baseline measurement before surgery. The authors concluded that postoperative PA’s discrimination of morbidity or mortality was superior to EuroSCORE II [35]. Next to a low PA (cut-off < 4.1°), reduced FFMI and increased extracellular water from BIA parameters can be used as predictors of prolonged hospital stay and in-hospital mortality (except FFMI). These observations were confirmed in a prospective cohort study of 50 patients undergoing cardiac surgery; a significant decrease in PA was observed between the preoperative stage and hospital discharge or three months postoperatively [41]. No difference according to the type of surgery was observed (coronary artery bypass grafting vs. valve replacement vs. combined procedure). A similar relationship to Stavrou et al. was observed for HGS. PA was associated with EuroSCORE and mechanical ventilation at all stages. The BIA measurements were performed before surgery, after 1 week, and after 3 months. Most of the patients showed recovery in muscle strength but persistent decreases in PA by 3 months post-surgery [38]. As postoperative care requires the extensive monitoring of various aspects, PA is a useful biomarker of recovery.

PA decline was also observed in long-term observations. In the prospective cohort study, which included 272 cardiac surgery patients, a year-long observation of PA changes showed reductions in PA values after surgery, with total recovery beginning at 6 months [37]. Moreover, age, combined surgery, and sex were predictors for PA reduction. PA impacted hospital LOS and ICU length of stay. Similar results were also observed for the handgrip strength test [37]. These results aligned with the observation in 195 patients undergoing cardiac surgery, where PA was positively correlated with the handgrip strength test and negatively with EuroSCORE II [36]. Moreover, the combination of PA and the handgrip strength test was associated with higher one-year all-cause mortality, prolonged ICU length of stay, and EuroSCORE II, compared to patients with higher PA and handgrip strength test results. The cut-off points were PA < 5.15 and HGS test < 25.5 for one-year all-cause mortality and PA < 5.15 and HGS test < 30.7 for prolonged ICU length of stay. The patients with low PA and handgrip strength were 9.3 times more likely to die and 4 times more likely to have prolonged ICU stays. As women generally had lower PA values and perioperative change was not so significant, female gender was identified as a protective factor in the multivariate analysis by the authors [36].

PA application for post-procedural monitoring was also shown in a pediatric population. In the cohort study in 122 children with congenital heart disease following cardiac surgery, a low PA (≤2.7° on day 2 after the procedure) was associated with longer pediatric intensive care unit length of stay [39]. This relationship was also present after adjustments for age, known risk factors, and length of surgery. The follow up of this observation was presented as a brief report from 100 pediatric patients with congenital heart disease undergoing cardiac surgery. The authors measured the preoperative proportion between PA at 200 kHz and PA at 5 kHz, and reported that it is associated with prolonged ICU stay [44].

The caliber of the procedure can potentially play a role, as PA assessed in 19 patients with increased surgical risk remained unchanged after transcatheter aortic valve implantation at 6-month follow-up [45].

Moreover, in patients with coronary artery disease who underwent CABG, PA was also reported as a factor of peak VO_2_ and anaerobic threshold in a cardiopulmonary exercise test [46]. The observation was performed in 33 patients; however, the authors did not provide information about the length of the follow-up or procedure details. Nonetheless, the results show the association between PA and physical performance in this group.

**Figure 2:**
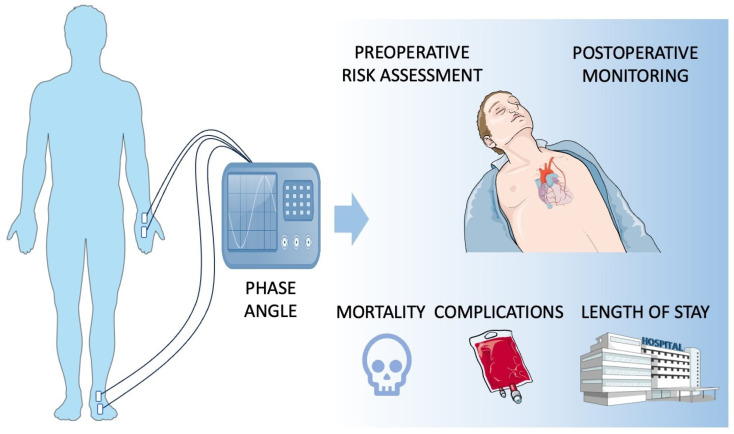
Summary of PA application in cardiac surgery patient assessment.

As presented above, PA can be used not only for pre-procedural risk stratification but also for post-procedural monitoring. Cardiac surgery—primarily extracorporeal circulation—causes serious stress to an organism, and PA enables the assessment of its regeneration at the cellular level in both the short and long term. A graphical summary of the abovementioned findings is presented in Figure 2.

### Limitations of the Available Literature

While PA derived from BIA has demonstrated substantial clinical potential in cardiac surgery, particularly for nutritional risk assessment and the prediction of adverse events, it is important to acknowledge certain methodological limitations in the available literature. The studies included in this review were based on a variety of BIA devices that differed in electrode configuration (bipolar vs. tetrapolar), electrode placement, and patient positioning (horizonal vs. vertical), all of which may influence PA measurements. Nevertheless, BIA devices are typically calibrated before measurement, which should partially mitigate device-related discrepancies [19]. Moreover, although PA measurement at 50 kHz is widely accepted as the clinical standard, some studies utilized alternative frequencies, and such methodological variability may also depend on the operator’s level of expertise. Importantly, as already mentioned, among all parameters derived from BIA, PA is considered the least susceptible to inter-device variability, as it reflects raw bioelectrical properties rather than being derived from proprietary algorithms used to estimate body composition.

PA is a valuable and widely used marker of cellular integrity and nutritional status, and it can be potentially dependent on individual characteristics such as age, sex, body mass, or ethnicity, which could support the application of PA in clinical patients’ assessment as it gathers and reflects all this information. However, for research purposes, adjustments for age, sex, and BMI could also be recommended, as they could potentially enhance the accuracy and interpretability of PA-based prognostic models.

In summary, these technical considerations do not diminish the prognostic value of PA but rather highlight the need for greater standardization of measurement protocols and calibration procedures. The establishment of international reference standards would enhance reproducibility and support the clinical integration of this promising biomarker in perioperative cardiac care.

## 6. Conclusions

PA is a parameter that can be used for nutritional status assessment in cardiac surgery patients. It is suggested that it is more accurate than other criteria in malnutrition identification. The available data shows its usefulness in preoperative risk assessment and post-procedural monitoring. PA could be introduced in everyday clinical assessment in cardiac surgery, but its validation is still to be confirmed in prospective randomized studies.

## Figures and Tables

**Table 1:** A summary of available studies that proved the usefulness of PA in cardiac surgery patients.

Reference	Size Group	PA Association	Study
Mortality			
[34]	A total of 204 patients after cardiac surgery with cardiopulmonary bypass	in-hospital mortality	Retrospective
[35]	A total of 179 cardiac surgery patients	in-hospital mortality	Prospective
[30]	A total of 277 patients undergoing major cardiac surgery	higher mortality at 1 month and at 12 months.	Prospective
[36]	A total of 195 patients undergoing cardiac surgery	higher one-year all-cause mortality	Prospective
Length of stay			
[34]	A total of 204 patients after cardiac surgery with cardiopulmonary bypass	prolonged hospital LOS	Retrospective
[30]	A total of 277 patients undergoing major cardiac surgery	prolonged hospital LOS	Prospective
[35]	A total of 179 cardiac surgery patients	prolonged hospital LOS	Prospective
[28]	A total of 325 cardiac surgical patients	prolonged ICU and hospital LOS	Prospective
[37]	A total of 272 cardiac surgery patients	prolonged ICU and hospital LOS	Prospective
[36]	A total of 195 patients undergoing cardiac surgery	prolonged ICU LOS	Prospective
[38]	A total of 127 well-nourished patients undergoing “on-pump” elective cardiac surgery	prolonged ICU LOS	Prospective
[39]	A total of 122 children with congenital heart disease following cardiac surgery	prolonged pediatric ICU LOS	Prospective
Complications			
[40]	A total of 168 elective cardiac surgical patients	longer cardiopulmonary bypass times	Prospective
[31]	A total of 342 low-operative-risk patients	postoperative morbidity	Prospective
[33]	A total of 642 adult patients undergoing elective cardiac surgery	blood transfusion	Retrospective
[37]	A total of 272 cardiac surgery patients	decline after procedure restored after 6 months	Prospective
[35]	A total of 179 cardiac surgery patients	significant impairment 7 days after the procedure	Prospective
[41]	50 patients undergoing cardiac surgery	mechanical ventilation	Prospective
[42]	114 older patients undergoing elective cardiovascular	predictor of hospital-acquired functional decline risk, undernutrition, and prolonged mechanical ventilation.	Prospective

ICU—intensive care unit, LOS—length of stay

## Abbreviations

The following abbreviations are used in this manuscript:

ACC	aortic cross-clamp
BIA	bioelectrical impedance analysis
BMI	body mass index
CABG	coronary artery bypass graft
CPB	cardiopulmonary bypass
CRP	C-reactive protein
ESPEN	european society for clinical nutrition and metabolism
ECW	extracellular water
FFMI	fat-free mass index
HGS	handgrip strength
ICU	intensive care unit
LVEF	left ventricle ejection fraction
LOS	length of stay
MUAC	mid-upper arm circumference
PA	phase angle
SMML	skeletal muscle mass loss
